# An investigation into the role of time-dependent cohesion in interseismic fault restrengthening

**DOI:** 10.1038/s41598-019-46241-5

**Published:** 2019-07-09

**Authors:** M. P. A. van den Ende, A. R. Niemeijer

**Affiliations:** 1 High Pressure and Temperature Laboratory, Department of Earth Sciences, Utrecht University, Utrecht, The Netherlands; 2 Present Address: Université Côte d’Azur, IRD, CNRS, Observatoire de la Côte d’Azur, Géoazur, France

**Keywords:** Geophysics, Geology

## Abstract

Earthquakes typically exhibit recurrence times that far exceed time-scales attainable in a laboratory setting. To traverse the temporal gap between the laboratory and nature, the slide-hold-slide test is commonly employed as a laboratory analogue for the seismic cycle, from which the time-dependence of fault strength may be assessed. In many studies it is implicitly assumed that all fault restrengthening emanates from an increase in the internal friction coefficient, neglecting contributions from cohesion. By doing so, important information is lost that is relevant for numerical simulations of seismicity on natural faults, as well as for induced seismicity. We conduct slide-hold-slide experiments on granular halite gouge at various normal stresses to assess the time-dependence of the internal coefficient of friction, and of the cohesion, independently of one another. These experiments reveal that both the internal friction coefficient and cohesion increase over time, but that these quantities do not share a common evolution, suggesting different underlying mechanisms.

## Introduction

Earthquakes are among the most disruptive of natural hazards known. Owing to their destructive potential and poor predictability, earthquakes and unstable frictional sliding in general receive considerable attention in laboratory, field, and modelling studies. For the assessment of seismic hazard, reliable estimates of fault strength and coseismic stress drop are of great importance. The rate at which a fault regains the frictional strength that was lost during a seismic event controls the recurrence time and the maximum strength that can be attained before the next earthquake^1,2^, and therefore many laboratory studies focus on the time-dependence of frictional strength^3–5^.

A major challenge that is inherent to laboratory work is the vast contrast in spatial and temporal scales associated with typical laboratory tests and natural seismic cycles. The recurrence time for natural earthquakes may vary from days up to many hundreds of years, and densification and restrengthening by relatively slow time-dependent processes (such as pressure solution)^6–9^ become significant over those time-spans. By contrast, the laboratory-scale equivalent of earthquakes, the so-called stick-slips^10^, commonly have interseismic periods (i.e. durations of the ‘stick’) of the order of several seconds to minutes, making a direct comparison between laboratory and natural stick-slip cycles non-trivial.

To bridge the temporal gap between natural and laboratory interseismic periods, the time-dependent strength recovery of faults is commonly studied by conducting *slide-hold-slide* (SHS) experiments^3^, in which the transient shear strength of the sample is monitored following a predetermined hold period. The difference between the peak and steady-state shear strength defines the restrengthening of the fault during the hold period (e.g.^4^), although additional measures have been proposed to quantify the restrengthening behaviour in more detail^11,12^. By systematically varying the duration of the hold, the rate of restrengthening of the material can be estimated, from which extrapolation to natural time-scales can be attempted (e.g.^1,13^).

In slide-hold-slide tests, the amount of restrengthening is typically reported as a change in apparent friction coefficient, Δ*μ*′ = Δ*τ*/*σ*_*n*_, implicitly assuming that cohesion is negligible in magnitude compared to the applied effective normal stress. However, several studies have suggested that cohesion cannot be neglected^14–17^, and thus needs to be considered separate from the internal coefficient of friction in the time-dependent strengthening of faults. To further investigate this, we conducted a suite of experiments in which fluid-rock interactions are significant at the time-scale of the experiment, and interseismic restrengthening is simulated. From these experiments, the time-dependence of the sample cohesion is estimated independently of the coefficient of friction.

Frictional restrengthening is referred to by many authors as ‘frictional healing’ or simply ‘healing’. To avoid confusion with the micro-scale process of grain boundary healing^18^, we shall consistently use the term ‘restrengthening’ to refer to the increase in mechanical resistance to shear deformation.

## Results

**Figure 1:**
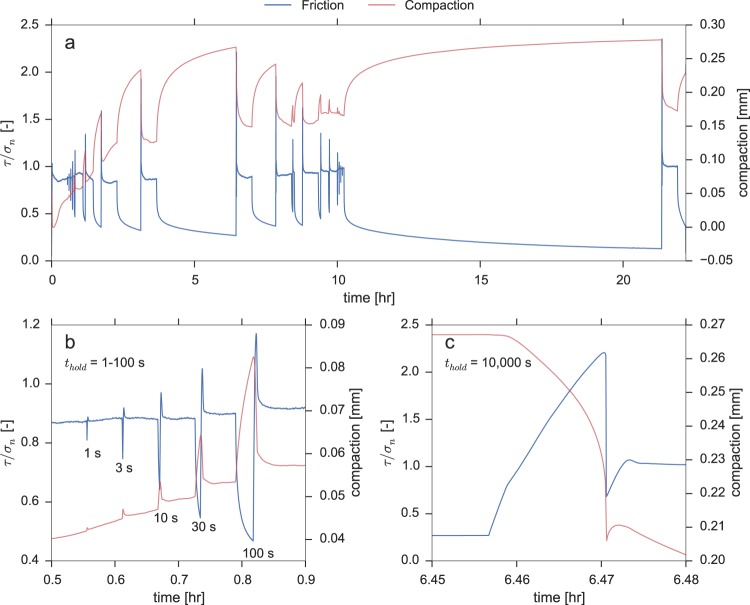
Mechanical results from experiment u473, conducted at a normal stress of 2.5 MPa. The blue lines display the apparent coefficient of friction, *τ*/*σ*_*n*_, the red lines display the axial displacement as recorded by the local LVDT, measuring compaction as positive. (**a**) Overview of all of the slide-hold-slide sequences conducted in this experiment; (**b**) Detailed view of the first five slide-hold-slides conducted. Note that resliding is stable; (**c**) Detailed view of the resliding conducted after a hold period of 10000 s. Note that resliding is unstable.

A representative overview of the shear stress and compaction data (measured by the local LVDT) that is typically recorded in the SHS tests is presented in Fig. 1. The steady-state frictional strength of the halite sample, reported as an apparent coefficient of friction (*τ*/*σ*_*n*_), is initially close to but slightly lower than 0.9, but over the duration of the experiment evolves to 1.0. Overall, the steady-state apparent friction coefficient fluctuates between 0.8 and 1.0, which may be related to an uneven load distribution of the torque cells due to a non-uniform thickness of the sample, or due to slight misalignment of the piston rings. As soon as active sliding is halted (i.e. the hold starts), the shear stress relaxes rapidly, accompanied by compaction of the sample. After the hold phase, when sliding is re-initiated, a clear peak stress is observed even for the shortest hold duration of 1 s, after which the stress evolves towards a new steady-state. During a reslide, the sample dilates until steady-state is reached. For relatively long hold durations (Δ*t* > 3000 s), we observe an overshoot in the stress drop as well as in sample dilatation following the peak stress (Fig. 1c), which is accompanied by a clearly audible acoustic emission. We will refer to this behaviour as ‘unstable’ resliding. Resliding after relatively short hold durations is nominally stable and does not show an overshoot in shear stress drop and dilatation (Fig. 1b), nor are audible acoustic emissions produced. The first occurrence of unstable sliding in each experiment is listed in Table 1.

**Table 1:** List of laboratory experiments with the applied normal stress, the range of hold durations, and indication of the hold step after which the first unstable slip event occurred.

Experiment	*σ*_*n*_ [MPa]	Hold steps [s]	Unstable after [s]
u472	5.0	1-3-10-30-100-300-1000-3000-10000	3000
u473	2.5	1-3-10-30-100-300-1000-3000-10000-3000-1000-300-100-30-10-3-1-40000	3000
u475	1.0	1-3-10-30-100-300-1000-3000-10000-3000-1000-300-100-30-10-3-1-40000	10000
u476	5.0	3000-10000-40000	10000
u480	5.0	10000-40000	—

Experiment u480 was unloaded prior to resliding to get a near-direct measurement of the cohesion.

**Figure 2:**
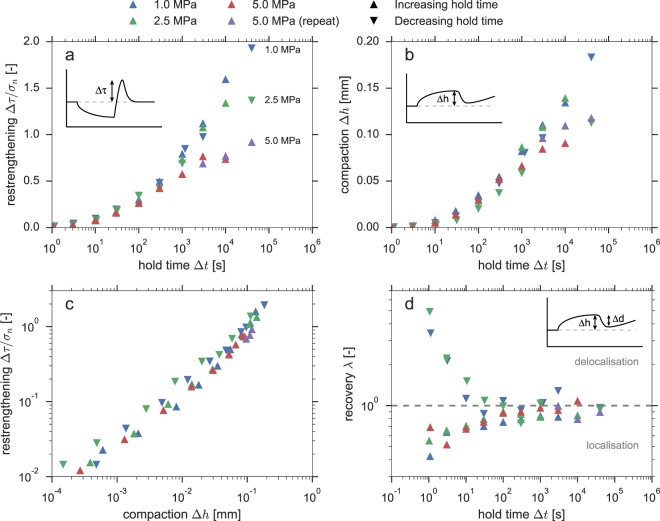
Overview of the mechanical response of the laboratory samples. (**a**) Frictional restrengthening, expressed as an apparent coefficient of friction Δ*μ*′ = Δ*τ*/*σ*_*n*_, as a function of hold duration; (**b**) Sample compaction during the hold as a function of hold duration; (**c**) Frictional restrengthening as a function of the compaction attained during the hold; (**d**) Volumetric strain recovery during resliding, *λ* = Δ*d*/Δ*h*, with values < 1 suggesting localised deformation, and values > 1 suggesting delocalisation. Values of *σ*_*n*_, and the direction of the slide-hold-slide sequence (increasing or decreasing hold steps) are as indicated in the legend.

The total restrengthening, expressed as an apparent coefficient of friction (Δ*μ*′ = Δ*τ*/*σ*_*n*_), does not obey a log-linear evolution with hold duration (Fig. 2a), nor can the data be represented by a power-law relation with a constant exponent (i.e. constant *β* in Δ*μ*′∝Δ*t*^*β*^). For short hold durations (Δ*t* < 1000), no effect of the imposed normal stress is observed, but for longer hold durations Δ*μ*′ increases faster with hold duration for lower *σ*_*n*_. By contrast, the total sample compaction achieved during each hold is not systematically related to the imposed normal stress (Fig. 2b). In addition, there exists a clear relation between the amount of restrengthening and the amount of compaction achieved during each hold (Fig. 2c).

Some first-order insight into the (possibly changing) degree of localisation of shear strain in the sample can be obtained from considering the volumetric deformation (compaction and dilatation). We define the recovery parameter *λ* as the ratio of the maximum dilatancy measured during resliding (Δ*d*) over the compaction attained during the hold period (Δ*h*), i.e. *λ* = Δ*d*/Δ*h* (see Fig. 2d and Methods section). For practically all of the resliding phases in the sequence of increasing hold duration (upward pointing triangles in Fig. 2d), only a portion of the compaction during the hold is recovered during resliding, which is indicated by *λ* < 1. This suggests that shear deformation is localised to some extent during these stages of the experiments, as the compaction achieved during the hold period (presumably occurring within the entire gouge layer) is not compensated by dilatant processes occurring in the (localised) actively sliding region. If shear deformation were fully distributed within the gouge layer and if the steady-state porosity during sliding at a constant velocity is constant, it is to be expected that between consecutive steady-states all of the compaction attained during the hold period is recovered upon resliding (*λ* = 1). The amount of nett compaction diminishes for longer hold durations, and turns to nett dilatation (*λ* > 1) for the sequence of decreasing hold duration (downward pointing triangles), suggesting progressive reworking (delocalisation) of the gouge layer. However, the longest hold duration of 40000 s that concludes the up-and-down sequence again shows near-neutral values of *λ* ≈ 1.

**Figure 3:**
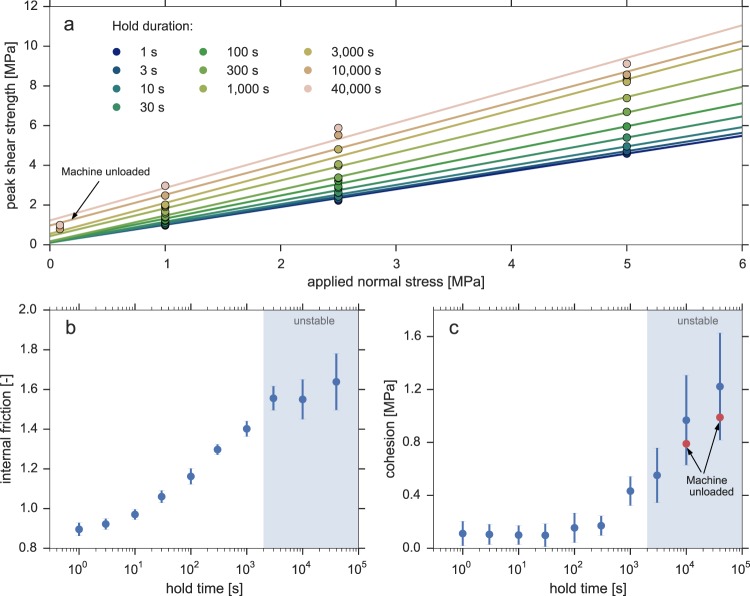
Analysis of the time-dependence of the internal friction coefficient and sample cohesion, as estimated by fitting a Mohr-Coulomb failure envelope to the data. The slope of this linear fit represents the internal friction coefficient, the intercept represents the cohesion. (**a**) Peak shear strength measured during resliding after each hold step, as a function of applied normal stress, and best-fit failure envelopes. Hold durations are indicated in the legend; (**b**) Internal coefficient of friction, as inferred from the slope of the failure envelopes in panel (a) as a function of hold time; (**c**) Sample cohesion, as inferred from the intercept of the failure envelopes as a function of hold time. Measurements done while the machine was unloaded are marked by the two red dots. Error bars indicate the standard error of the parameter estimates resulting from the regression. The shaded area indicates the region of hold durations after which resliding has been oberved to be unstable.

To estimate the amount of cohesion that was attained over time, we construct Mohr-Coulomb failure envelopes for each individual hold phase (see Methods and Fig. 3). The slope of this envelope represents the internal coefficient of friction, and its intercept represents the sample cohesion. Overall, the data at relatively short hold durations (Δ*t* < 1000 s) can be captured well with a linear failure envelope (Fig. 3a). For longer hold durations, the scatter of the data relative to the linear failure envelope increases, which is reflected by the uncertainty of the estimates of the internal friction and cohesion in Fig. 3b,c. At this point, it is unclear whether this is purely due to experimental variability, or due to a breakdown of the assumed linear Mohr-Coulomb model for these long hold durations (e.g. due to stress-dependent time evolution of friction and/or cohesion).

When looking more closely to the inferred internal friction (Fig. 3b), we find that it increases non-linearly with the logarithm of the hold duration. For relatively short hold durations (<3000 s), the evolution is slightly concave-upwards, but for longer hold durations it plateaus (within the uncertainty of the estimate). Conversely, for hold durations <1000 s, the inferred cohesion (Fig. 3c) is small compared to the frictional strength and near-constant in time, but increases rapidly for longer hold durations. The uncertainty of the estimate is relatively large for these longer hold durations, but the measurements made when the machine was unloaded (see Methods) fall within the range of uncertainty and support the observed trend. Since the data obtained from unloading the machine are independent of the assumed Mohr-Coulomb failure model, it justifies our choice for a linear failure envelope.

## Discussion

### Comparison with previous work

The rate of frictional restrengthening observed in slide-hold-slide experiments conducted on (wet) granular halite is generally of the order of 0.1–0.3 decade^−1^ (e.g.^5,7^), being at least one order of magnitude faster than quartz at similar conditions^5,9^. Our results display rates falling in a similar range, from 0.25 decade^−1^ for hold durations 1000 s or a normal stress of 5 MPa, and up to 0.5 decade^−1^ for hold durations 1000 s and a normal stress of 1 MPa (see Fig. 2). The rate of restrengthening cannot be captured by a single log-linear relation. Rather, the data suggest either a bi-linear (e.g.^5,9^) or power-law evolution (e.g.^13,19^). Furthermore, the applied normal stress (*σ*_*n*_) seems to have no effect on the restrengthening behaviour for hold durations <1000 s. For hold durations longer than 1000 s, samples subjected to lower *σ*_*n*_ show faster apparent restrengthening.

At first, this behaviour appears counter-intuitive, considering that all samples display a similar amount of compaction achieved for a given hold phase, as measured by the local LVDT (Fig. 2b). Since densification has been positively correlated to gouge strength^20,21^, it is to be expected that all samples show a similar degree of restrengthening for each hold duration. However, by expressing the frictional restrengthening in terms of an apparent friction coefficient (Δ*τ*/*σ*_*n*_), it is implicitly assumed that cohesion is negligible. When this assumption is violated, the contribution of cohesion (*C*_*s*_) to the total shear strength is underestimated, and this underestimation becomes more severe with increasing normal stress:

1 $$\mu ^{\prime} =\frac{\mu {\sigma }_{n}+{C}_{s}}{{\sigma }_{n}}=\mu +\frac{{C}_{s}}{{\sigma }_{n}}$$

Therefore, experiments performed under low *σ*_*n*_ will receive a larger contribution from cohesion to the apparent friction coefficient, and will thus appear to strengthen faster for a given rate of increase of *C*_*s*_. The strengthening rate observed in experiments performed at various *σ*_*n*_ start to diverge after a hold duration of 1000 s, which coincides with the onset of increasing *C*_*s*_. The timing of this onset may be related to the time that is required to form cohesive contacts.

### Degree of localisation

The recovery of volumetric strain (*λ*) as calculated for the laboratory experiments, shows a decreasing tendency for nett compaction with increasing experiment duration. During a given hold phase, both the active (localised) and spectator regions in the gouge will densify, but during resliding only the active region will dilate to restore the steady-state porosity that was previously attained within this region. A value of *λ* < 1 accordingly indicates that shear deformation is localised, and that compaction in the spectator regions is dominant over the volumetric strain of the active regions in the gouge. As the spectator regions densify and compaction slows down in the approach to zero porosity, *λ* tends to 1 (no nett compaction). Moreover, when the volume of gouge that contributes to shear deformation increases (i.e. if shear delocalisation occurs), then gouge dilation exceeds the near-zero compaction of the spectator region, so that *λ* > 1.

In this view, we can interpret the volumetric strain behaviour as observed in the laboratory experiments. During initial stages of the experiments, shear deformation is localised and the spectator region compacts at a substantial rate compared to the volumetric strains in the active region, so that $\lambda \ll 1$. Over the course of the experiment, the spectator region densifies and compaction decelerates, resulting in an increase in *λ*, approaching a neutral value of 1. During the decreasing hold duration sequence, reworking of the gouge leads to *λ* > 1. These interpretations are, however, merely exhibitive. For a number of resliding phases, no steady-state sample thickness is achieved over the duration of resliding, with the sample still dilating at the moment that the next hold phase is commenced, underestimating *λ*. If a longer duration of resliding was chosen, the resulting trends in *λ* may have been different. Regardless, the frictional response of the sample (e.g. Δ*τ*) does not show any effects of sliding history, as similar values are obtained for both the increasing and decreasing hold duration sequences (Fig. 2a), which indicates that the frictional strength is insensitive to the evolution of localised regions in the gouge.

### Mechanisms for time-dependent restrengthening

In previous studies it has been inferred that pressure solution and granular flow are both dominant deformation mechanisms in halite aggregates subjected to the experimental conditions imposed in this study^22–24^. These two mechanisms have been closely considered by^25^ and^26^, and are described by a microphysical model framework which we will refer to as the *Chen-Niemeijer-Spiers* (“CNS”) model. In short, the CNS model assumes that the imposed deformation is fully accommodated by parallel operation of dilatant granular flow, and non-dilatant pressure solution creep. The competition between these mechanisms controls the overall microstructural state (porosity) of the gouge, and produces a wide range of frictional behaviour (see e.g.^27,28^). We will interpret the outcomes of the experiments in this framework.

First of all, we consider the time-dependence of the internal coefficient of friction (*μ*). In the CNS model, the strength of an aggregate is controlled by its porosity through the average dilatancy angle *tan ψ* as^25,26^:

2 $$\mu =\frac{\tilde{\mu }+\,\tan \,\psi }{1-\tilde{\mu }\,\tan \,\psi }$$

where $\tilde{\mu }$ is the coefficient of friction of a single grain-grain contact. For the purpose of this discussion, $\tilde{\mu }$ can be taken to be constant. The dilatancy angle describes the amount of dilatation per unit shear displacement associated with neighbour swapping and grain sliding, and is a common notion to soil mechanics^29,30^. During a hold period, the gouge undergoes time-dependent compaction by pressure solution, through which *tanψ* increases. As a result, the overall internal friction of the aggregate increases with time, giving rise to the time-dependence of the macroscopic internal friction. The maximum value of *μ* is achieved when the porosity reaches zero (or a small minimum value). This further implies a limit to the internal coefficient of friction that can be attained during a seismic cycle, if gouge compaction is the only restrengthening mechanism.

It is important to realise that in the above discussion, the intrinsic grain boundary friction coefficient ($\tilde{\mu }$) is taken to be constant. This is in strong contrast to previous notions that the time-dependence of fault strength stems from an intrinsic elevation of asperity contact strength through plastic creep or asperity welding^31–33^. In the view of *adhesion theory of friction* (and rate-and-state friction by association^34^), the internal coefficient of friction at a macroscopic scale is directly related to the ‘quality’ of micro-scale asperity contacts. In our laboratory experiments, it cannot be excluded that the intrinsic friction of the grain-grain contacts increases in a manner that is envisioned by adhesion theory. However, in Discrete Element Method simulations of stick-slip cycles^35^ in which the grain contact friction is taken to be constant, fault restrengthening is entirely attributed to compaction of the aggregate (by pressure solution). Time-dependence of contact strength is therefore not a requirement to explain the time-dependence of the macroscopic internal friction coefficient.

While gouge densification accounts for the time-dependence of the internal coefficient of friction, it does not directly account for the observed time-dependence of cohesion. We propose that surface energy-driven growth of grain contacts or contact asperities (‘islands’) results in cementation of grain contacts, generating grain contact-scale cohesion. The operation of contact growth and island growth has been observed in numerous experiments^36–40^, and has been theoretically analysed by^18^. This mechanism conceptually shares similarities with the aforementioned adhesion theory: the intrinsic shear strength of an individual grain contact increases by growth of contact area. However, in this context contact and island growth is interpreted to contribute to sample cohesion rather than to internal friction. Moreover, the mechanism by which island growth is assumed to operate (fluid-assisted diffusive mass transfer; e.g.^18,37,38^) is notably different from plastic creep, which is the dominant mechanism considered in adhesion theory^33^. Lastly, following the interpretation of^18^ (supported by laboratory observations reported by e.g.^36^), nett island growth impedes pressure solution creep by constriction of the channels through which excess dissolved material diffuses into the pore space (i.e. impedance of the serial process of dissolution-diffusion-precipitation associated with pressure solution). This introduces a competition between time-dependent strengthening through compaction by pressure solution on the one hand, and strengthening through cohesion generated by island growth on the other. By comparing the trends in Fig. 3b,c, it is observed that the rate of increase in the internal friction coefficient diminishes at around the moment in time that the inferred cohesion increases substantially. While this correlation between the dynamics of the two quantities is somewhat circumstantial, the interplay between the two aforementioned processes (pressure solution and island growth) warrants further investigation in future studies.

Although both the internal friction coefficient and cohesion contribute to the overall shear strength of a material, it is important to distinguish between these two components when the magnitude of the effective normal stress is relevant for the problem that is considered. While at high effective normal stress the contribution of friction exceeds that of cohesion, cohesion may play a dominant role in the strengthening under relatively low effective normal stress conditions, such as shallow focus earthquakes. The stress drop associated with (induced) earthquakes in e.g. geological reservoirs may receive a significant contribution from cohesion. Moreover, the magnitude of the *in-situ* fluid pressure is generally poorly constrained^41^, and various lines of evidence suggest that the fluid pressure may approach lithostatic values in fault settings (e.g. subduction zones) in which the total stress is high^42–45^. Under near-lithostatic fluid pressure conditions, a contribution of cohesion of the order of several MPa becomes significant in determining the absolute strength and stress drop on the fault. Lastly, cohesion may exert a strong influence on the velocity- or slip-dependent stability of a fault (as seen in Fig. 3), as the fault slip required to remove grain-scale cohesion (i.e. the weakening distance) may be much shorter than for purely frictional weakening. To assess whether or not the development of cohesion is efficient under *in-situ* fault conditions, it is important to consider the specific micro-mechanisms, of which the relevance in nature can be assessed independently of the laboratory results.

### Implications for the seismic cycle

We now follow the interpretations above to discuss the restrengthening and stability of natural faults. During the interseismic period fault slip rates are relatively low, and so the fault compacts by pressure solution creep and increases its strength. Simultaneously, far-field tectonic loading increases the stress on the fault. The instability (earthquake or slow slip event) occurs when the stress supported by the fault exceeds its strength, after which the fault accelerates, dilates, and weakens. For a given tectonic loading rate, the recurrence time and stress drop are therefore controlled by the rate of compaction (and by the rate of cohesion development).

This leaves now three possible scenarios: in the first scenario, compaction and fault strengthening are relatively slow (compared to the tectonic loading rate). The gouge compacts during the interseismic period, but does not reach its minimum (near-zero) porosity before the next seismic event. This means that the fault continuously strengthens over time, but at a rate that, on the long-term, is lower than the tectonic stressing rate. This is a scenario that is typically considered in studies employing rate-and-state friction, where the state parameter (usually denoted by *θ*) and corresponding fault strength are allowed to increase indefinitely, but at a log-linear rate that is slower than the linear stressing rate.

Based on the results presented here, as well as those by previous studies^5,13,19^, it cannot be excluded that faults strengthen more rapidly than the stressing rate, e.g. when considering a power-law relation with a time-exponent *n* > 1. In this second scenario, a limit to the maximum attainable fault strength (c.f.^13^) is required for an earthquake to recur on a given fault patch. For granular gouges, this limit corresponds to a fully densified (i.e. minimum porosity) microstructural state, with additional grain cementation. The failure strength of a fully densified gouge must still remain at or below the failure strength of the surrounding host rock in order to achieve failure within the gouge itself (i.e. the fault must be weak relative to its surroundings^44^). A scenario of rapid restrengthening is increasingly more appropriate for faults segments at greater depth, as aggregate compaction and strengthening rates generally increase with increasing temperature and pressure^9,16,46^. It further implies that the recurrence time is one-sidedly controlled by the tectonic loading rate, as the strength of the fault has reached a constant value well before the failure stress is reached.

Lastly, a third scenario can be considered where fluid-rock interactions are slow, but tectonic loading is virtually absent (such as in an intraplate setting). In this situation, a fault remains unperturbed for an extended period of time (e.g. millions of years), over which it acquires a given amount of shear strength. If the state of stress around the fault is perturbed, for example by human subsurface activities of injection or extraction of fluids, then an instability may be triggered when the stress supported by the fault exceeds its current strength. However, whether or not this instability is succeeded by subsequent events would depend on the present-day rate of restrengthening relative to the perturbed loading rate. In other words, reactivation of faults that have been inactive for long geological time periods may not necessarily lead to repeated seismic activity of similar intensity. The question of prolonged induced seismicity may be addressed by considering the strength of the fault prior to reactivation, as well as the present-day strengthening rates at *in-situ* conditions. Furthermore, the stress drop associated with fault reactivation likely includes loss of cohesion, which is often neglected in numerical studies of fault reactivation. To assess how much cohesion contributes to the total shear strength of a material, laboratory failure tests should be performed (e.g.^47^).

The scenarios above illustrate the relevance of explicitly considering the micro-mechanics of friction. On the basis of commonly used rate-and-state friction or linear slip weakening formulations, it cannot be assessed what the maximum strength of a fault will be, or how the fault will behave after reactivation. By relating the overall mechanics of a fault to the relevant micro-scale processes, hypotheses and predictions may be formulated that can be tested in a field or laboratory setting. In this study, granular flow, pressure solution, and nett asperity growth were thought to constitute the overall mechanical response of the aggregate, but other mechanisms (e.g. microcracking, mineral precipitation) could be treated in a similar fashion, and provide insight into the restrengthening behaviour of a particular fault of interest. Adopting appropriate constitutive relations for the microphysical processes then allows for a quantitative analysis of the seismic cycle and coseismic stress drop based on physical principles (e.g.^28^).

## Conclusions

In this work, we report a suite of slide-hold-slide laboratory experiments conducted on granular halite, from which we inferred the time-dependence of the internal coefficient of friction and of the sample cohesion independently. This was done by treating the second sliding phase as a Mohr-Coulomb failure process, and measuring the peak stress as a function of the imposed normal stress. In these experiments, we found that the frictional restrengthening, measured as an increase of the apparent coefficient of friction (i.e. Δ*μ*′ = Δ*τ*/*σ*_*n*_), increases non-linearly with the logarithm of hold time, showing a concave-up strength evolution in a semi-logarithmic representation. For hold durations >1000 s, the restrengthening rates seem to decrease with increasing applied normal stress, which alludes to the presence of cohesion. Furthermore, both the internal friction coefficient and sample cohesion increase with the duration of the hold phase, contributing to the overall frictional restrengthening. However, these two quantities do not share a common trend. The internal coefficient of friction increases rapidly at first, but levels off for hold durations over 3000 s. By contrast, the sample cohesion is inferred to be negligible for hold durations <1000 s, with a rapid increase for longer durations. As a result the total shear strength (i.e. *τ* = *μσ*_*n*_ + *C*_*s*_) continues to increase with time. These results indicate that the contribution of cohesion to the total fault strength cannot be excluded, especially when long interseismic time periods and hydrothermal conditions are considered.

## Methods

### Description of apparatus

**Figure 4:**
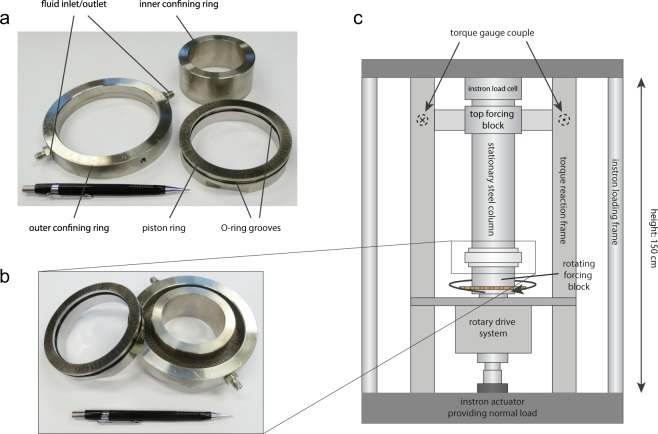
Overview of the sample assembly and apparatus used. (**a**) Photograph of one piston ring and the inner- and outer stainless steel confining rings. The pen for scale has a length of 14.5 cm; (**b**) Photograph of partially assembled sample. The gouge is deposited on the piston ring that is confined by the stainless steel rings (right). The second ring (left) is placed on top of the gouge to close off the assembly; (**c**) Schematic diagram of the rotary shear apparatus.

All of the experiments reported in this study were conducted at ambient conditions in a rotary shear apparatus (Fig. 4), located at the High Pressure and Temperature laboratory in Utrecht, the Netherlands. In this apparatus, a 1 mm thick granular sample of synthetic gouge is enclosed by two ring-shaped toothed pistons with an inner and outer diameter of 80 mm and 100 mm respectively. The piston teeth are closely spaced and have a groove depth of 0.1 mm, and are oriented perpendicular to the direction of sliding. This artificial roughness inhibits slip on the interface between the steel and the gouge, and promotes distributed deformation. Localisation of slip, if any, occurs within the body of the gouge, rather than on the interface between the gouge and the steel^24^. Stainless steel inner and outer rings confine the sample radially to prevent extrusion of the gouge. The outer ring features two diametrically placed pore-fluid ports to allow for saturation of the sample at atmospheric fluid pressure. Inner and outer O-rings, fitted in between the pistons and the inner and outer confining rings, prevent loss of pore fluid and sample material. The sample assembly is gripped between two cylindrical forcing blocks, positioned within an Instron 1362 loading frame. The axial (normal) load applied by this frame is measured by a 100 kN Instron load cell and servo-controlled with a precision of ~10 N, which corresponds to 3.5 kPa of normal stress on the sample.

After application of the normal stress, the bottom forcing block is rotated with respect to the top block at a computer-controlled angular velocity by a motor-driven gear box. The range of velocities that could be attained is 1–1000 μm/s with the gear configuration used in these experiments, and the total shear displacement is in principle unlimited. The shear resistance supported by the sample as a result of shear deformation is measured by two load cells with a resolution of ~10 kPa that are mounted in a torque couple connected to the top forcing block. Angular displacements are measured by a potentiometer with a resolution of 1 μm geared to the bottom of the rotating bottom block, and axial displacements (i.e. dilatation/compaction of the sample) are measured by two Linear Variable Differential Transformers (LVDTs), one situated at the base of the loaded column (100 mm full scale, 1 μm resolution) and one in between the top and bottom forcing blocks (1 mm full scale, 0.1 μm resolution; referred to as ‘local LVDT’). See also^22,24,48^. Data was acquired by a National Instruments BNC connector block (model BNC-2111) at a rate of 1000 Hz, but down-sampled to an effective rate of 1–900 Hz, which was varied manually during the experiment to capture rapid changes in the data stream (e.g. during changes in the driving velocity).

### Sample material and preparation

Since it is expected that fluid-rock interactions play a dominant role in the frictional restrengthening of natural gouges at depth, we use analytical-grade (99.8% pure) halite (NaCl; VWR Chemicals BV, prod. no. 27810.364) for sample material. Owing to its high solubility (0.163 m^3^/m^3^ at 21 °C), halite has previously been used as an analogue material for quartz at hydrothermal conditions (e.g.^22–24^). The sample material was manually crushed using a mortar and pestle, and sieved to a grain size <75 μm to obtain the desired starting grain size. The gouge was carefully distributed over the bottom piston ring with a small spatula, and the layer was levelled with a smooth levelling ring. The inner and outer steel ring walls were cleaned of any remaining gouge before the top piston ring was put into place, closing off the assembly. The initial porosity was estimated by carefully weighing the sample mass and measuring the thickness of the assembly. However, during the experiment, sample material intruded into the small annular cavities between the pistons and the inner and outer confining rings, up to the level of the O-rings. Although the material did not extrude from the assembly itself, the material loss from between the pistons was sufficient to introduce a significant error in the estimate of the porosity during the experiments. Hence, we only report values of axial displacements (i.e. compaction or dilatation) relative to the start of shear deformation, which are independent of our estimates of the sample mass still present between the forcing blocks.

The pore fluid was prepared by dissolving an excess of halite in de-mineralised water. The brine was allowed to equilibrate for an hour at a temperature of 50 °C, and was slowly cooled back to room temperature. This ensured that the brine was fully saturated, or slightly over-saturated. The brine was then contained in a 100 ml syringe, which could be readily attached to the pore-fluid system of the sample assembly. Over time, small amounts of fluid evaporated out of the syringe, producing a slight over-saturation, as evidenced by the small halite crystals forming at the bottom of the syringe.

### Laboratory procedure

The sample assembly was loaded axially to a target load corresponding to a sample normal stress of 1, 2.5 or 5 MPa, which was varied between the different experiments. To mature the gouge and to reach a steady-state porosity more rapidly, the gouge was first sheared ‘dry’ (at 40–70% RH) over a distance of 20 mm at 10 μm/s. Then, the pore-fluid was introduced by a syringe pump at a rate of 0.5 ml/min until sample saturation was achieved, after which the sample was allowed to compact for 1 hour. One of the fluid ports remained open to the atmosphere, as to maintain drained conditions while the sample compacted. Shear deformation was then re-initiated by sliding at a velocity of 10 μm/s over 20 mm of shear displacement, followed by a slide-hold-slide (SHS) sequence.

For each individually tested normal stress, a similar SHS sequence was conducted with hold durations ranging from 1 up to 40000 s (~11 hr), in increments of half an order of magnitude in duration. To test for sliding history effects, the SHS sequence was repeated by first step-wise increasing the hold duration from 1 s up to 10000 s (increasing hold steps), and then reversing the sequence back down to 1 s (decreasing hold steps; see Table 1). Finally, the longest hold duration of 40000 s was performed once at the end of the sequence. Between each hold phase the sample was deformed at a sliding velocity of 10 μm/s until a steady-state shear stress was achieved, which could take up to several minutes for some of the longer hold durations. The first experiment in this series, u472, conducted at 5 MPa normal stress, suffered from a technical failure after the first 10000 s hold step. We consider the data from all subsequent sliding phases compromised and these are not included in the analyses presented in this work. To compensate for the partial loss of data, a repeat experiment was conducted (u476) with hold steps of 3000, 10000, and 40000 s. Since the effect of sliding history is not immediately apparent for the observed restrengthening (see Fig. 2), it is assumed that, especially for longer hold durations, the procedure adopted here erases any history effects, and so the data from experiment u476 are taken to be representative. The reproducibility of the 3000 and 10000 s hold steps at 5 MPa (red and purple triangles in Fig. 2) further attests to this.

In addition to the procedure described above, we conducted one experiment (u480) at 5 MPa with hold durations of 10000 and 40000 s, in which the machine was completely unloaded prior to each reslide. This was done by first rotating the bottom forcing block back until all shear stress was removed, before removing the applied normal stress (similar to the hold-slide approach of^15^). The residual weight of the cross-head and spacer components that was supported by the sample was 0.38 kN (0.13 MPa on the sample). Then, the sample was deformed at a rate of 10 μm/s in the absence of any normal stress applied by the loading frame, so that the measured peak strength gave a near-direct measurement of the sample cohesion (shear strength in the absence of normal stress). Owing to the apparatus design, no shear deformation of the sample could occur during back-rotation other than elastic unloading, i.e. sliding on the sample was not reversed. Because of this, it is unlikely that any sample cohesion was removed by the unloading procedure, provided that the cohesive strength was sufficient to sustain elastic unloading and re-loading. Note that during the hold periods of u480, the sample was kept under the same loading conditions as the other experiments (i.e. constant normal stress, and a slowly relaxing shear stress), and that the load was manually removed only after each hold phase was concluded.

### Data analysis

#### Time-dependence of friction and cohesion

To assess the contribution of cohesion to the overall strength of the gouge, the following data analysis procedure was adopted: first, it was assumed that during resliding following a given hold duration, the sample ‘failed’ macroscopically at the peak shear strength. Since the experiments were conducted at various normal stresses, Mohr-Coulomb failure envelopes of the form *τ*_*peak*_ = *μσ*_*n*_ + *C*_*s*_ could be constructed by plotting the peak shear strength *τ*_*peak*_ of the sample after a given hold duration against the applied normal stress *σ*_*n*_. This was done for all normal stresses employed in the experiments, and subsequently for each hold duration. The internal coefficient of friction *μ* and the sample cohesion *C*_*s*_ could then be obtained though linear regression. Ordinary least-squares regression was performed by the Python StatsModels package^49^. This procedure was adopted for each hold duration individually, so that the time-evolution of *μ* and *C*_*s*_ could be inferred. The values of the cohesion inferred in this manner are compared to those obtained from unloading the machine (experiment u480), as to investigate the possibility that the sample failure envelope becomes nonlinear approaching the origin (e.g.^50^), for which a linear failure criterion would not be an accurate description.

#### Localisation of shear strain

It is commonly found in laboratory friction tests that shear strain localises within a narrow region, often up to several tens of μm in thickness^24,51,52^. Even though post-mortem microstructural analysis may readily reveal any localisation features present in the sample, it is non-trivial to establish the timing of such features, and to relate the recorded mechanical response to microstructural developments, particularly when the sample experienced a complex sliding history (e.g. a multi-step slide-hold-slide sequence). Nonetheless, the tendency for localisation may be identified from the mechanical data by considering the volumetric strain response of the sample. When granular flow is confined to a narrow region in the sample, the volumetric response of the bulk of the sample will be predominantly compactive, as (slow) compaction in a wide bulk region outweighs dilatant granular flow in a narrow localised region. Conversely, a continuous dilatant response hints to progressive reworking of the gouge (delocalisation), as progressively larger volumes of the gouge participate in dilatant granular flow. If shear deformation were fully distributed within the gouge layer, it is to be expected that between consecutive steady-states all of the compaction attained during the hold period is recovered upon resliding. Although this approach does not reveal the degree of localisation in absolute terms, it provides a first-order insight into the partitioning of volumetric strain upon resliding.

To quantify the tendency for localisation in the laboratory experiments, we define the volumetric strain recovery parameter *λ* as:

3 $$\lambda =\frac{{\rm{\Delta }}d}{{\rm{\Delta }}h}$$

where Δ*h* is the compaction measured at the end of the hold phase (relative to the start of the hold phase), and Δ*d* is the maximum dilatancy measured from the initiation of resliding up to the start of the next hold phase (see also inset in Fig. 2d), so that *λ* represents the relative amount of compaction during the hold that is recovered during resliding. Values of *λ* < 1 indicate nett compaction (not all porosity loss is recovered), which suggests that deformation is localised. A value of *λ* > 1 indicates nett dilatation, likely caused by reworking of dense regions of the gouge. A value of *λ* = 1 indicates that all porosity loss during the hold is recovered.

## Data Availability

All data are available from the corresponding author upon request.
